# GPNMB-Positive Cells in Head and Neck Squamous Cell Carcinoma—Their Roles in Cancer Stemness, Therapy Resistance, and Metastasis

**DOI:** 10.3389/pore.2022.1610450

**Published:** 2022-08-19

**Authors:** Yohei Kawasaki, Hitomi Suzuki, Shinsuke Suzuki, Takechiyo Yamada, Maya Suzuki, Ayumi Ito, Haruka Hatakeyama, Masahito Miura, Yasufumi Omori

**Affiliations:** ^1^ Department of Otorhinolaryngology and Head-and-Neck Surgery, Akita University Graduate School of Medicine, Akita, Japan; ^2^ Department of Molecular and Tumour Pathology, Akita University Graduate School of Medicine, Akita, Japan

**Keywords:** epithelial-mesenchymal transition, cancer stem cells, radiotherapy, head and neck squamous cell carcinoma, glycoprotein nonmetastatic melanoma protein B, prognostic factor

## Abstract

**Objective:** Despite the use of surgical and chemoradiation therapies, head and neck squamous cell carcinoma (HNSCC) still has a poor prognosis. Immune checkpoint inhibitors have been shown to prolong life expectancy but have limited efficacy. Glycoprotein nonmetastatic melanoma protein B (GPNMB) has received significant attention in breast cancer treatment, in which it has been associated with cancer stem cells (CSCs) and epithelial-mesenchymal transition (EMT); however, the function of GPNMB in HNSCC is completely unknown. This study aimed to clarify the characteristics of GPNMB-positive cells *in vitro* and their association with the prognosis by immunostaining clinical specimens.

**Methods:** We examined the sphere formation, invasion, and migration ability of GPNMB-positive cells in four HNSCC cell lines *in vitro*. We also immunostained biopsy specimens with GPNMB from 174 patients with HNSCC diagnosed, treated, and followed-up in our institution to evaluate overall survival and progression-free survival.

**Results:** GPNMB-positive cells showed enhanced sphere formation, invasion, and migration, suggesting that they could have CSC characteristics and the ability to induce EMT, as reported for breast cancer. Clinical specimens showed that overall survival was 39.4% and 57.8% (*p* = 0.045) and that progression-free survival was 27.6% and 51.6% (*p* = 0.013) for the high-expression and the low-expression groups, respectively, indicating poor prognosis for the high GPNMB group. The high GPNMB group was also more resistant to chemoradiation and bioradiotherapy. GPNMB was more highly expressed in metastatic lymph nodes than in the primary tumor.

**Conclusion:** GPNMB-positive cells might have CSC characteristics and induce EMT. Detailed functional analyses of GPNMB in HNSCC and the establishment of therapies targeting GPNMB will lead to improved prognoses.

## Introduction

Head and neck squamous cell carcinoma (HNSCC) is the sixth most common cancer worldwide, with 600,000 new cases each year (1, 2). Patients with HNSCC often have advanced cancer at the time of diagnosis. Even after surgery and chemoradiation, recurrence is common, with a 5-year survival rate of approximately 50% (3). Developing new therapeutic targets and treatment methods to improve the prognosis of HNSCC is therefore important.

Glycoprotein nonmetastatic melanoma protein B (GPNMB) is a type I transmembrane protein that has been reported to be involved in various processes, including cell differentiation, inflammation, tissue regeneration, and cell migration (4, 5). Thus, although GPNMB is known to play various roles in normal tissues, it has also been reported to be overexpressed in malignant tumors including glioma (6), hepatocellular carcinoma (7), and rectal cancer (8). The prognosis of these cancers is poor when overexpressed, and GPNMB promotes invasion and metastasis in malignant melanoma (9, 10). Recently, cells highly expressing GPNMB have attracted considerable attention in breast cancer. These cells have the characteristics of cancer stem cells (CSCs) and display a phenotype of epithelial-mesenchymal transition (EMT) (11). CSCs are, in general, capable of self-renewal, differentiation, tumorigenesis, and resistance to drugs and radiation due to their ability to enter a dormant state and the abundant expression of drug exporters. EMT is an essential process in embryonic development and tissue repair, as well as in cancer invasion and metastasis. The EMT-related transcription factors such as SNAIL and TWIST, and TGF-β signaling are often associated with the acquisition of CSC properties in breast epithelial cells (12). Chen et al. (13) stated that CSCs are therefore considered to be the root cause of cancer metastasis and recurrence and that therapeutic targeting of EMT-related molecules is a promising therapeutic strategy to eradicate CSCs and concluded that GPNMB is an essential protein for EMT and CSC gene expression. In other words, GPNMB is also a CSC marker, which induces EMT and promotes metastasis and invasion. To inhibit cancer recurrence and metastasis, GPNMB-targeted therapy should be established. In fact, the *in vivo* knockdown of GPNMB has successfully reduced tumorigenicity (11).

In the case of HNSCC, the function of GPNMB is largely unknown, and although there have been recent reports that GPNMB overexpression is a factor associated with a poor prognosis (14) and that GPNMB promotes migration (15), there have been few reports of GPNMB in HNSCC. We hypothesized that cells expressing GPNMB in HNSCC could have CSC characteristics and induce EMT, leading to metastasis. If confirmed, we will be able to establish a completely new treatment complementary to surgical therapy, radiation therapy, and immune checkpoint inhibitors. In this study, we investigated the sphere formation, invasion, and migration ability of isolated GPNMB-positive cell population *in vitro*. We also immunostained clinical specimens to investigate the relationship with overall survival (OS) and progression-free survival (PFS).

## Materials and Methods

### Cell Culture

We used four HNSCC cell lines: HO-1-u-1 (floor of the mouth), Sa3 (gingiva), HSC2 (oral cavity), and HSC4 (tongue). For a monolayer culture, these lines were maintained in Roswell Park Memorial Institute (RPMI) 1640 medium (Nissui, Tokyo, Japan) supplemented with 10% fetal bovine serum (Clontech Laboratories, Mountain View, CA, United States), 2 mM L-glutamine, 2.4 g/L sodium bicarbonate, 100 U/ml penicillin, and 100 μg/ml streptomycin. In culture conditions, we incubated the cells at 37°C in a humidified atmosphere containing 5% carbon dioxide in air. SCH772984 (Selleck Chemicals, Houston, TX, United States), an ERK inihibitor, was added to the medium at 5 μM for some experiments.

### Flow Cytometry Analysis and Cell Sorting

For the analysis of the GPNMB-positive fraction, we incubated cell pellets with allophycocyanin (APC)-conjugated anti-human GPNMB monoclonal antibodies (303822) (Novus Biologicals, Littleton, CO, United States) at a dilution of 1:11 at 4°C for 15 min. After washing with phosphate-buffered saline three times, we added 2 μg/ml propidium iodide to exclude dead cells. The cells were then filtered through a 40-μm cell strainer (Falcon, USA) and subjected to a FACSAria III cell sorter (BD, Osaka, Japan). To determine the negative fraction, we used APC-conjugated mouse IgG2b isotype antibodies (Novus Biologicals).

### Sphere Formation Assay

We employed the cell sorter to separate the cells into GPNMB-positive and GPNMB-negative fractions. Serum-free semisolid medium was constituted by adding 0.33% agar to 3D Tumorsphere Medium XF (PromoCell GmbH, Heidelberg, Germany). We placed 1 × 10^3^ scattered cells in 80 μl of the serum-free semisolid medium on 100 μl of solidified serum-free RPMI1640 basal layer containing 0.5% agar in each well of a 96-well plate and cultured them for 20 days. Forms of more than 50 cells were counted as spheres.

### Cell Migration and Invasion Assay

For the migration assay, we placed an 8-μm pore filter in a cell culture insert (BD Biosciences Discovery Labware, Bedford, MA, United States) in the lower compartment of a 24-well plate containing 1 ml of serum-loaded RPMI 1640 medium. We seeded 5.0 × 10^3^ cells suspended in serum-free RPMI 1640 medium in each upper well. After 48 h of incubation, the upper wells were swabbed and stained with hematoxylin-eosin to count the number of cells.

For the invasion assay, we precoated the 8-μm pore filters of the cell culture inserts with 500 μg of Matrigel (BD Biosciences Pharmingen), dried them in an incubator for 24 h, and rehydrated them in RPMI 1640 medium for 1 h before inoculating the cells. This assay was performed according to the same protocol as the migration assay described above, except that the cells were incubated for 72 h (16).

### Patients

The study examined 174 patients diagnosed with HNSCC who were treated and followed up at Akita University from January 2010 to December 2019 (Table 1). Computed tomography, magnetic resonance imaging, and positron emission tomography-computed tomography were employed to determine the clinical staging, which followed the Union for International Cancer Control criteria. Eighty-six patients underwent chemoradiotherapy with cisplatin or bioradiotherapy with cetuximab (Table 2), and 88 patients underwent surgery (Table 3). The mean observation period was 31.6 months (range: 2–60 months). Informed consent was obtained from all patients, and the study was approved by the Akita University Ethics Committee and conducted in compliance with the Declaration of Helsinki.

**TABLE 1:** Patient characteristics. All patients.

Characteristic		Values
Age (years)		65.4 ± 10.6
Sex (male/female)		144/30
T stage	T1	16 (9.2%)
	T2	71 (40.8%)
	T3	37 (21.3%)
	T4	50 (28.7%)
N stage	N0	49 (28.1%)
	N1	17 (9.8%)
	N2	104 (59.8%)
	N3	4 (2.3%)
M stage	M0	174 (100.0%)
Stage	I	11 (6.3%)
	II	27 (15.5%)
	III	19 (10.9%)
	IV	117 (67.3%)
Tumor sites	Tongue	32 (18.4%)
	Nasopharynx	9 (5.1%)
	Oropharynx	55 (31.6%)
	Hypopharynx	64 (36.9%)
	Gingiva	14 (8.0%)

**TABLE 2:** Patient characteristics. Patients who underwent chemoradiotherapy or bioradiotherapy.

Characteristic		Values
Age (years)		65.6 ± 9.1
Sex (male/female)		76/10
T stage	T1	6 (7.0%)
	T2	36 (41.9%)
	T3	21 (24.4%)
	T4	23 (26.7%)
N stage	N0	13 (15.1%)
	N1	12 (13.9%)
	N2	57 (66.3%)
	N3	4 (4.7%)
M stage	M0	86 (100.0%)
Stage	I	2 (2.3%)
	II	7 (8.1%)
	III	12 (14.0%)
	IV	65 (75.6%)
Tumor sites	Tongue	2 (2.3%)
	Nasopharynx	9 (10.5%)
	Oropharynx	40 (46.5%)
	Hypopharynx	33 (38.4%)

**TABLE 3:** Patient characteristics. Patients who underwent surgery.

Characteristic		Values
Age (years)		65.6 ± 9.1
Sex (male/female)		68/20
T stage	T1	10 (11.4%)
	T2	35 (39.8%)
	T3	16 (18.2%)
	T4	27 (30.7%)
N stage	N0	36 (40.9%)
	N1	5 (5.7%)
	N2	47 (53.4%)
	N3	0 (0.0%)
M stage	M0	88 (100.0%)
Stage	I	9 (10.2%)
	II	20 (22.7%)
	III	7 (8.0%)
	IV	52 (59.1%)
Tumor sites	Tongue	30 (34.1%)
	Nasopharynx	0 (0.0%)
	Oropharynx	15 (17.0%)
	Hypopharynx	31 (35.2%)
	Gingiva	12 (13.6%)

### Ethical Approval

Informed consent was obtained from all patients. All procedures used in this research were approved by the Ethics Committee of Akita University Hospital (Approval Number: 2532).

### Immunostaining and Evaluation Methods

Immunohistochemical staining was performed on paraffin sections using the polymer-peroxidase method. Briefly, the 3-μm thick sections were deparaffinized and rehydrated routinely. Antigen retrieval was performed by boiling the sections in 0.01 M citrate buffer (pH 6.0) for 40 min. After the sections were rinsed in distilled water, the endogenous peroxidase was inactivated with 3.0% hydrogen peroxide in distilled water for 10 min at room temperature. After rinsed in Tris-buffered saline (TBS), pH 7.4, the sections were incubated with 10% normal goat serum for blocking for 60 min at room temperature and then reacted with a primary antibody of interest for 90 min at room temperature. After a wash in TBS, the secondary antibody (EnVision+/HRP; DakoCytomation, Glostrup, Denmark) was added, and the sections were incubated for 60 min at room temperature. To visualize positive signals, the peroxidase reaction was performed using 0.02% 3,3′-diaminobenzidine tetrahydrochloride (DAB) (Sigma-Aldrich, St. Louis, MO, United States) for 3 min at room temperature. The sections were counterstained with Mayer’s hematoxylin, then dehydrated and mounted. The following primary antibodies were used: rabbit polyclonal anti-GPNMB antibody (1:50, 20338-1-AP, Proteintech, Rosemont, IL, United States), rabbit monoclonal anti-SOX2 antibody (1:100, ab93689, Abcam, Cambridge, United Kingdom), rabbit monoclonal anti-Nanog antibody (1:300, ab109250, Abcam), and rabbit polyclonal anti-SNAIL + SLUG antibody (1:900, ab93689, Abcam). The intensity was assessed by at least three board-certified pathologists. The evaluation method was classified by the percentage of GPNMB-positive tumor cells, which were negative, weak, moderate, and strong according to Rietbergen et al. (17); ≤10% of the stained tumor cells were negative (0); 11%–25% were weak (1); 25%–50% were moderate (2); and ≥50% were strong (3). Negative and weak were categorized as the low group, and moderate and strong as the high group.

### Statistical Analysis

All experiments were independently repeated at least three times. We employed the Wilcoxon signed-rank test to compare the intensity of the primary lesions and lymph node metastases. Survival was estimated using the Kaplan–Meier method, and statistical significance was tested using log-rank tests. We used Student’s t-test to perform the data analysis and estimate the statistical significance; *p*-values less than 0.05 were considered statistically significant (*: *p* < 0.05, **: *p* < 0.01, ***: *p* < 0.001, ****: *p* < 0.0001). We performed a multivariate analysis using a Cox proportional hazards model if a significant difference was observed. IBM SPSS version 21 was used for the statistical processing (IBM Inc., Armonk, NY, USA).

## Results

### Positive Rate of GPNMB in Head and Neck Squamous Cell Carcinoma Cell Lines

**FIGURE 1:**
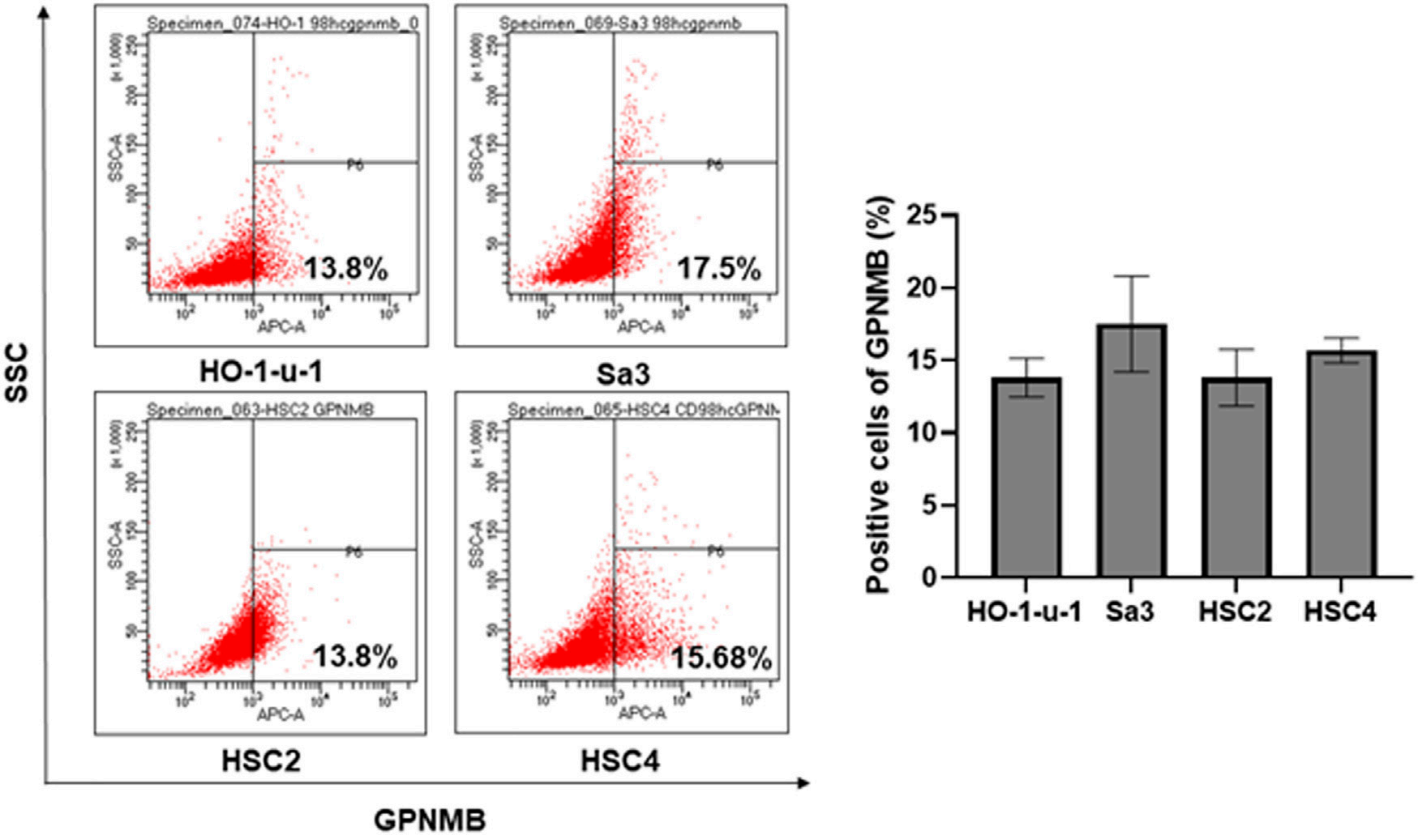
Percentage of GPNMB-positive cells in HNSCC cell lines. The upper left is HO-1-u-1 (floor of the mouth cancer), the upper right is Sa3 (gingival cancer), the lower left is HSC2 (oral cavity cancer), and the lower right is HSC4 (tongue cancer). When the positivity rate was examined by flow cytometry, the positivity rate of GPNMB was approximately 15%.

The positive rate of GPNMB in the HNSCC cell lines was 13.8% for HO-1-u-1, 17.5% for Sa3, 13.8% for HSC2, and 15.68% for HSC4 (Figure 1). No reports have examined the positivity of GPNMB in HNSCC by flow cytometry or in other carcinomas. In HNSCC cell lines, the GPNMB positivity rate is expected to be approximately 15%.

### GPNMB-Positive Cells Show Increased Capacity for Sphere Formation

**FIGURE 2:**
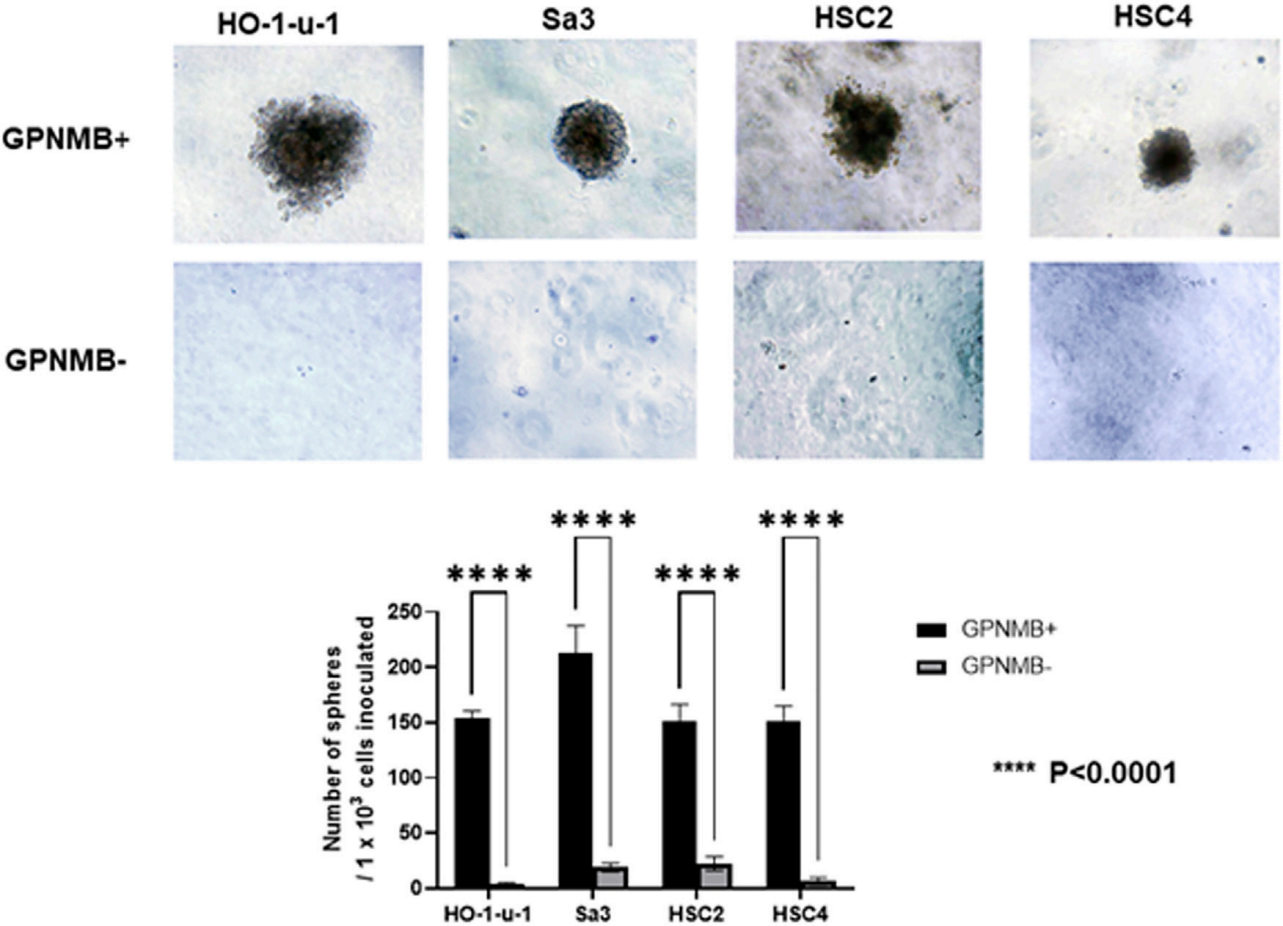
GPNMB-positive cells can form spheres in serum-free semisolid medium. Using a flow cytometer, the cells were separated into GPNMB-positive and GPNMB-negative cells and were cultured in serum-free semisolid medium. The top of the figure shows GPNMB-positive cells, and the bottom shows GPNMB-negative cells. GPNMB-positive cells could form a large number of spheres (*p* < 0.0001).

Similar to normal tissue stem cells, when CSCs are cultured in serum-free medium on non-attachment dishes or in serum-free semisolid medium, they proliferate as spherical cell aggregates and maintain an undifferentiated state without maturing into non-CSCs, allowing for pure CSC cultures (18, 19). We isolated GPNMB-positive and GPNMB-negative cells separately by FACS and cultured them in serum-free semisolid medium for 20 days. The GPNMB-negative cells demonstrated little ability to form spheres, whereas the GPNMB-positive cells showed an efficient ability to form a large number of spheres. There is the possibility that GPNMB-positive cells have CSC characteristics (Figure 2).

### GPNMB-Positive Cells Have Increased Invasive and Migratory Potential

**FIGURE 3:**
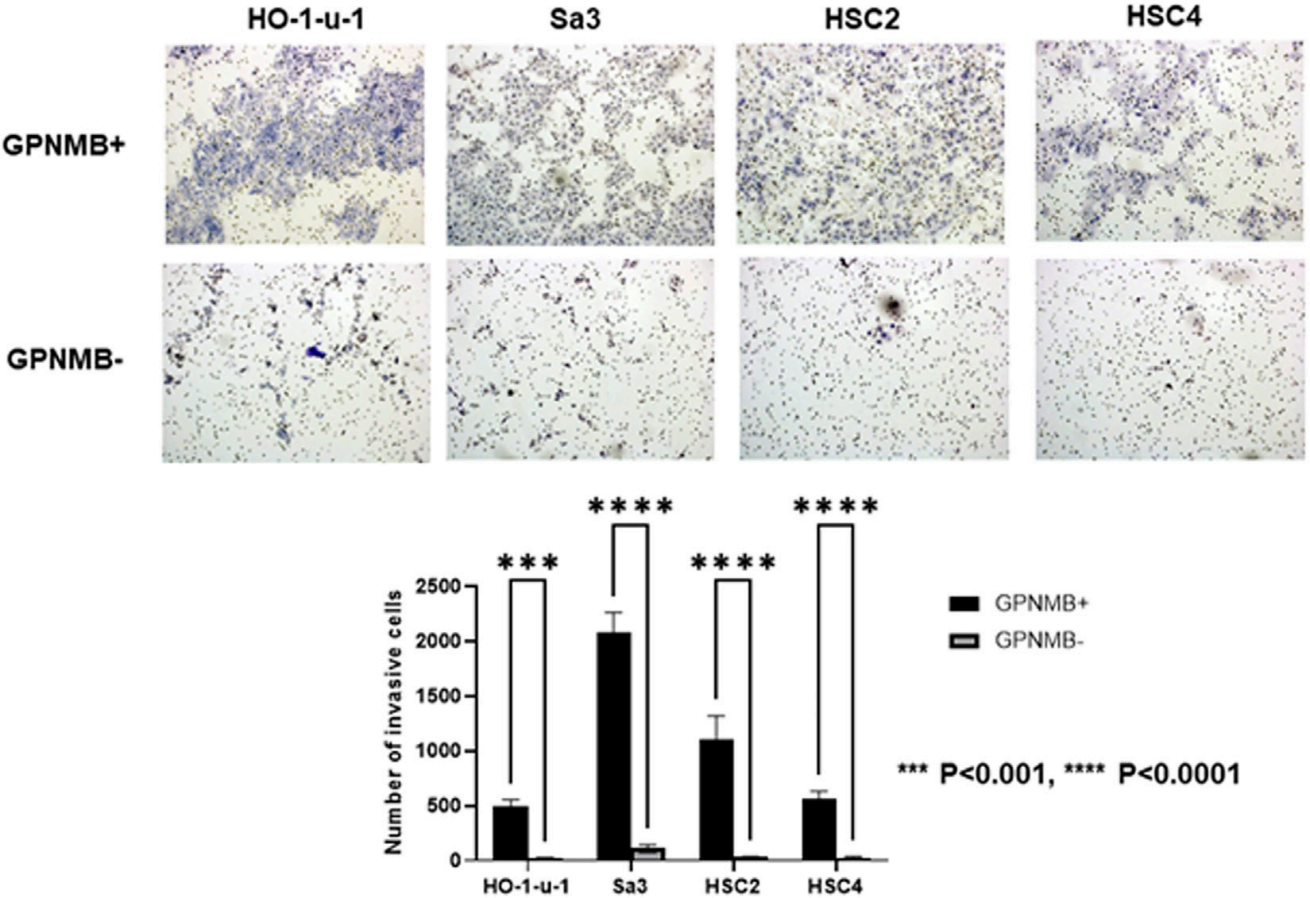
GPNMB-positive cells have an increased invasive potential. Using a flow cytometer, the cells were separated into GPNMB-positive and GPNMB-negative cells. The Boyden chambers were coated with Matrigel to examine their invasive ability. GPNMB-positive cells showed enhanced invasive ability (*p* < 0.0001).

**FIGURE 4:**
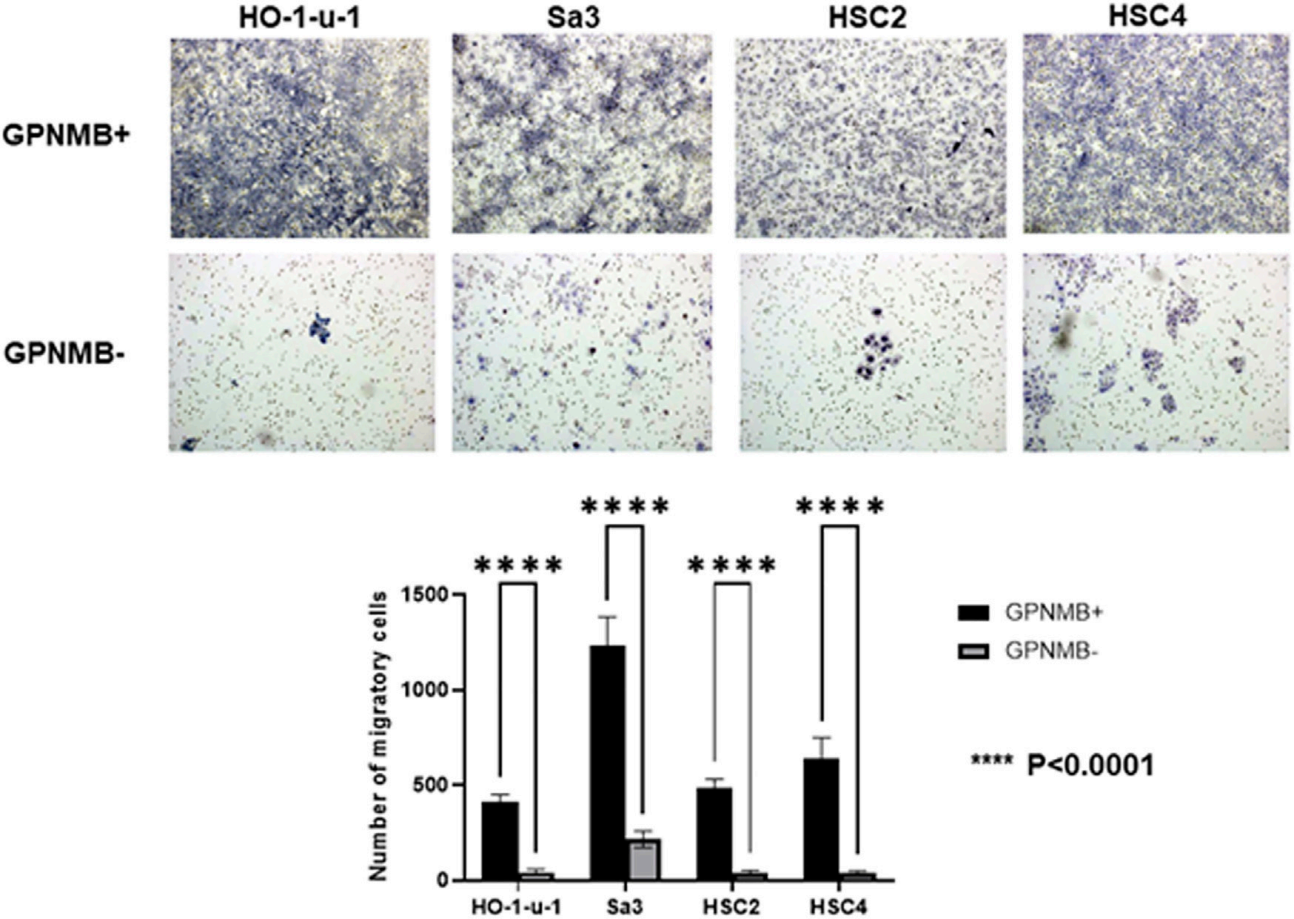
GPNMB-positive cells have an increased migratory ability. GPNMB-positive and GPNMB-negative cells were placed in a Boyden chamber, and their migratory ability was examined. The GPNMB-positive cells had enhanced migration ability (*p* < 0.0001).

We investigated the role of GPNMB in invasion and migration. Compared with the GPNMB-negative cells, the GPNMB-positive cells had greatly enhanced invasive and migratory potential (Figures 3, 4). It has been reported that GPNMB-positive cells can induce EMT (20); our result is consistent with that report.

### Relationship Between GPNMB Expression and Overall Survival/Progression-Free Survival

**FIGURE 5:**
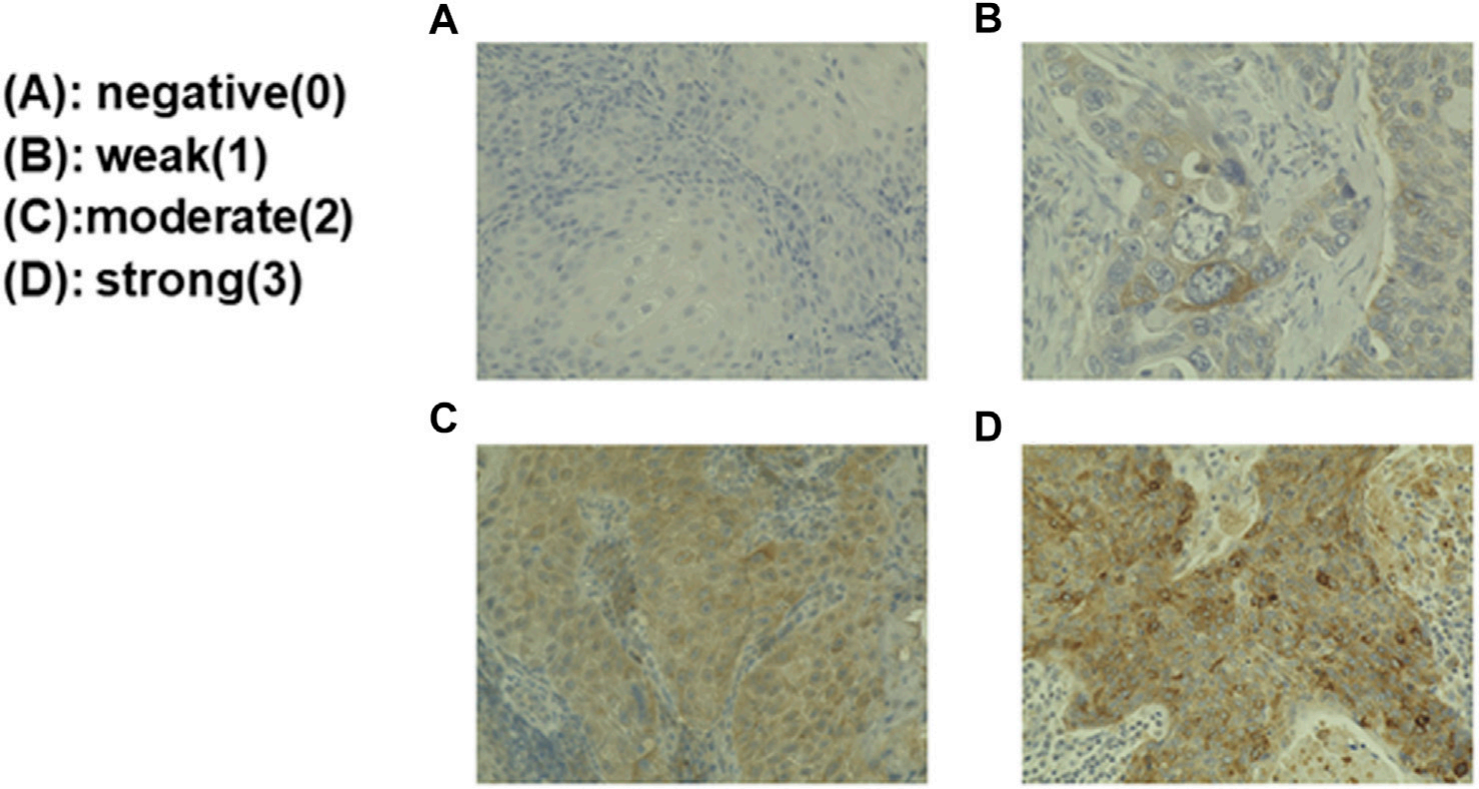
Biopsy specimens from the patients with HNSCC were immunostained and the staining intensity classified. Biopsy specimens were immunostained with anti-GPNMB antibody. The upper left is GPNMB-negative **(A)**; the upper right is weakly positive GPNMB **(B)**; the lower left is moderately positive GPNMB **(C)**; and the lower right is strongly positive GPNMB **(D)**. The evaluation was performed by board-certified pathologists.

**FIGURE 6:**
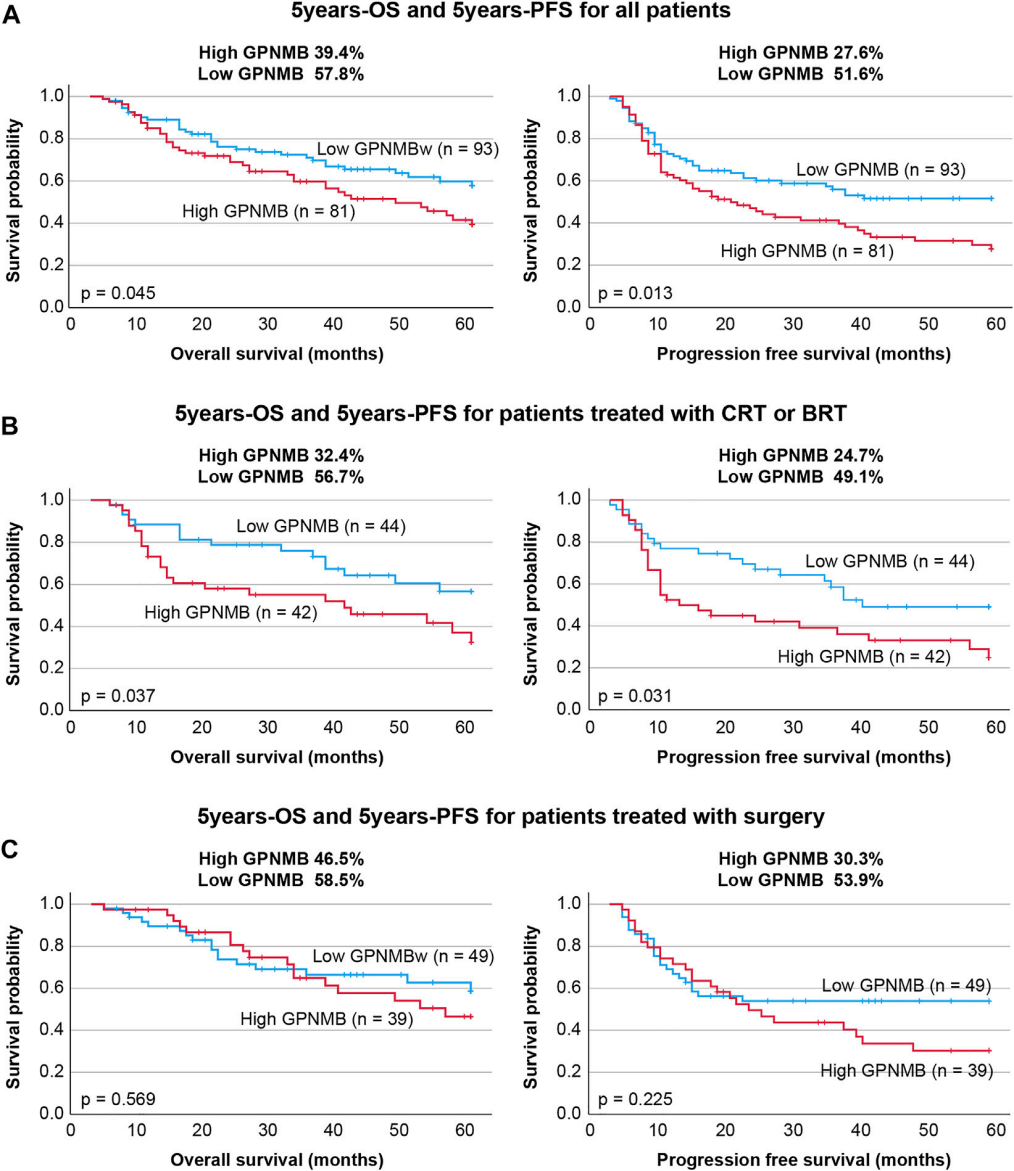
Comparison of GPNMB expression level with OS and PFS. **(A)** The OS and PFS and GPNMB expression levels of all patients were examined. The high GPNMB expression group had significantly lower OS and PFS. **(B)** We studied 86 patients treated with chemoradiotherapy or bioradiotherapy and examined their GPNMB expression levels. Both OS and PFS were significantly lower in the high-expression group, who were also radioresistant. **(C)** In the surgery group, no difference in OS and PFS was observed in the intensity of GPNMB expression.

The biopsy specimens from patients with HNSCC were immunostained with anti-GPNMB antibody. Negative and weak were categorized as the low group, whereas moderate and strong were categorized as the high group (Figure 5). We classified 81 of the 174 patients into the high GPNMB expression group and 93 into the low GPNMB expression group and examined their OS (Figures 6A–C, left) and PFS (Figures 6A–C, right). The OS of the high and low-expression groups was 39.4% and 57.8%, respectively (*p* = 0.045). The PFS of the high and low-expression groups was 27.6% and 51.6%, respectively (*p* = 0.013). The low-expression group showed a predominantly good OS and PFS (Figure 6A). We performed univariate and multivariate analyses, which showed that the independent prognostic factors for OS were tumor node metastasis (TNM) stage (hazard ratio [HR] 1.874, 95% CI 1.139–3.084, *p* = 0.013) and GPNMB (HR 1.722, 95% CI 1.075–2.758, *p* = 0.024) (Table 4). The only independent prognostic factor for PFS was GPNMB (HR 1.680, 95% CI 1.110–2.545, *p* = 0.014) (Table 5).

**TABLE 4:** Univariate and multivariate analysis for overall survival of all patients.

		Univariate analysis			Multivariate analysis		
		HR	95% CI	*p*	HR	95% CI	*p*
Age	(<65 vs. ≥65 years)	1.507	0.944–2.406	0.086	1.758	1.088–2.841	0.021
Sex	(Female vs. Male)	1.340	0.688–2.610	0.390	1.204	0.606–2.389	0.596
T stage	(T1-2 vs. T3-4)	1.989	1.242–3.183	0.004**	1.874	1.139–3.084	0.013*
N stage	(N0 vs. N1-3)	1.818	1.031–3.206	0.039*	1.697	0.920–3.130	0.091
Stage	(I-II vs. III-IV)	1.860	0.892–3.875	0.098			
GPNMB	(Low vs. High)	1.588	1.004–2.513	0.048*	1.722	1.075–2.758	0.024*

HR, hazard ratio; CI, confidence interval; GPNMB, glycoprotein nonmetastatic melanoma protein B; *p < 0.05; **p < 0.01.

**TABLE 5:** Univariate and multivariate analysis for progression-free survival of all patients.

		Univariate analysis			Multivariate analysis		
		HR	95% CI	*p*	HR	95% CI	*p*
Age	(<65 vs. ≥65 years)	1.028	0.688–1.536	0.894	1.144	0.754–1.736	0.527
Sex	(Female vs. Male)	1.418	0.774–2.596	0.259	1.251	0.670–2.336	0.482
T stage	(T1-2 vs. T3-4)	1.530	1.021–2.294	0.040*	1.510	0.985–2.317	0.059
N stage	(N0 vs. N1-3)	1.488	0.924–2.396	0.102	1.336	0.797–2.240	0.271
Stage	(I-II vs. III-IV)	1.241	0.704–2.188	0.455			
GPNMB	(Low vs. High)	1.650	1.102–2.472	0.015*	1.680	1.110–2.545	0.014*

HR, hazard ratio; CI, confidence interval; GPNMB, glycoprotein nonmetastatic melanoma protein B; *p < 0.05; **p < 0.01.

### GPNMB is Associated With Sensitivity to Chemoradiotherapy and Bioradiotherapy

In this study, we classified the 86 patients treated with chemoradiotherapy or bioradiotherapy into either a high GPNMB expression group (42 patients) or a low GPNMB expression group (44 patients) and examined the OS and PFS. The OS of the high and low-expression groups was 32.4% and 56.7%, respectively (*p* = 0.037). The PFS of the high and low-expression groups was 24.7% and 49.1%, respectively (*p* = 0.031) (Figure 6B). The results of the univariate and multivariate analyses showed that the prognostic factors for OS were TNM classification (HR 3.374, 95% CI 1.629–6.986, *p* = 0.001) and GPNMB (HR 2.843, 95% CI 1.445–5.592, *p* = 0.002) (Table 6). The prognostic factors for PFS were also TNM classification (HR 2.209, 95% CI 1.177–4.143, *p* = 0.014) and GPNMB (HR 2.136, 95% CI 1.172–3.813, *p* = 0.013) (Table 7). GPNMB expression was closely related to radiosensitivity.

**TABLE 6:** Univariate and multivariate analysis for overall survival of the patients who underwent chemoradiotherapy or bioradiotherapy.

		Univariate analysis			Multivariate analysis		
		HR	95% CI	*p*	HR	95% CI	*p*
Age	(<65 vs. ≥65 years)	1.507	0.944–2.406	0.086	1.811	0.901–3.640	0.095
Sex	(Female vs. Male)	1.340	0.688–2.610	0.390	1.423	0.415–4.878	0.575
T stage	(T1-2 vs. T3-4)	1.989	1.242–3.183	0.004**	3.374	1.629–6.986	0.001**
N stage	(N0 vs. N1-3)	1.818	1.031–3.206	0.039*	1.344	0.485–3.724	0.569
Stage	(I-II vs. III-IV)	1.860	0.892–3.875	0.098			
GPNMB	(Low vs. High)	1.931	1.023–3.642	0.042*	2.843	1.445–5.592	0.002**

HR, hazard ratio; CI, confidence interval; GPNMB, glycoprotein nonmetastatic melanoma protein; *p < 0.05; **p < 0.01.

**TABLE 7:** Univariate and multivariate analysis for progression-free survival of the patients who underwent chemoradiotherapy or bioradiotherapy.

		Univariate analysis			Multivariate analysis		
		HR	95% CI	*p*	HR	95% CI	*p*
Age	(<65 vs. ≥65 years)	1.028	0.688–1.536	0.894	1.213	0.660–2.227	0.534
Sex	(Female vs. Male)	1.418	0.774–2.596	0.259	1.952	0.585–6.512	0.277
T stage	(T1-2 vs. T3-4)	1.530	1.021–2.294	0.040*	2.209	1.177–4.143	0.014*
N stage	(N0 vs. N1-3)	1.488	0.924–2.396	0.102	1.241	0.503–3.063	0.640
Stage	(I-II vs. III-IV)	1.241	0.704–2.188	0.455			
GPNMB	(Low vs. High)	1.842	1.039–3.264	0.036*	2.136	1.172–3.893	0.013*

HR, hazard ratio; CI, confidence interval; GPNMB, glycoprotein nonmetastatic melanoma protein; *p < 0.05; **p < 0.01.

### Relationship Between GPNMB and Surgical Treatment

There was no significant difference in OS (*p* = 0.569) or PFS (*p* = 0.225) among the 39 patients in the high-expression group and the 49 patients in the low-expression group (Figure 6C).

### GPNMB Expression Was Stronger in Metastatic Lymph Nodes Than in the Primary Tumor

**FIGURE 7:**
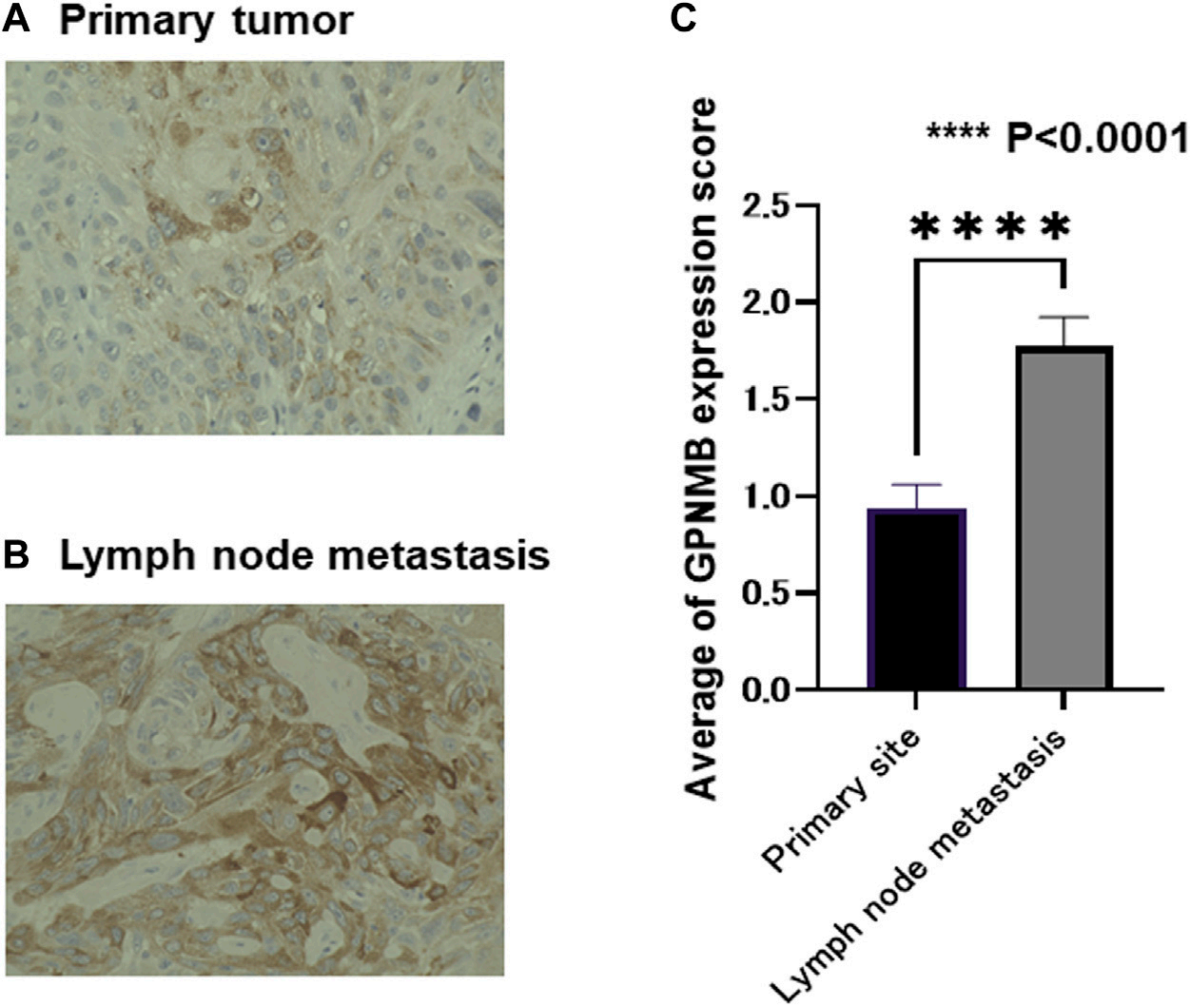
Metastatic lymph nodes express a larger amount of GPNMB than the primary tumor. In the 47 patients with lymph node metastasis, we compared GPNMB expression levels in the primary tumor and lymph node metastasis of matched patients. The evaluation method was the same as in Figure 5. Representative micrographs are presented **(A,B)**. The evaluation was performed by board-certified pathologists. The metastatic lymph nodes had a larger amount of GPNMB compared with the primary tumor (*p* < 0.0001) **(C)**.

In the surgical group, 47 patients were pathologically found to have metastatic lymph nodes. The intensity of GPNMB in the biopsy specimens and metastatic lymph nodes was evaluated from 0 to 3. In these 47 patients, the standard errors of the primary tumor and lymph nodes were compared. The intensity of GPNMB was higher in the metastatic lymph nodes than in the primary tumor (*p* < 0.0001) (Figure 7). From a pathological viewpoint, it can be assumed that GPNMB is closely related to metastasis. We used the Wilcoxon signed-rank test for analysis.

### Correlation of the Expression Levels of GPNMB With Those of Cancer Stem Cell Markers and Epithelial-Mesenchymal Transition Markers

**FIGURE 8:**
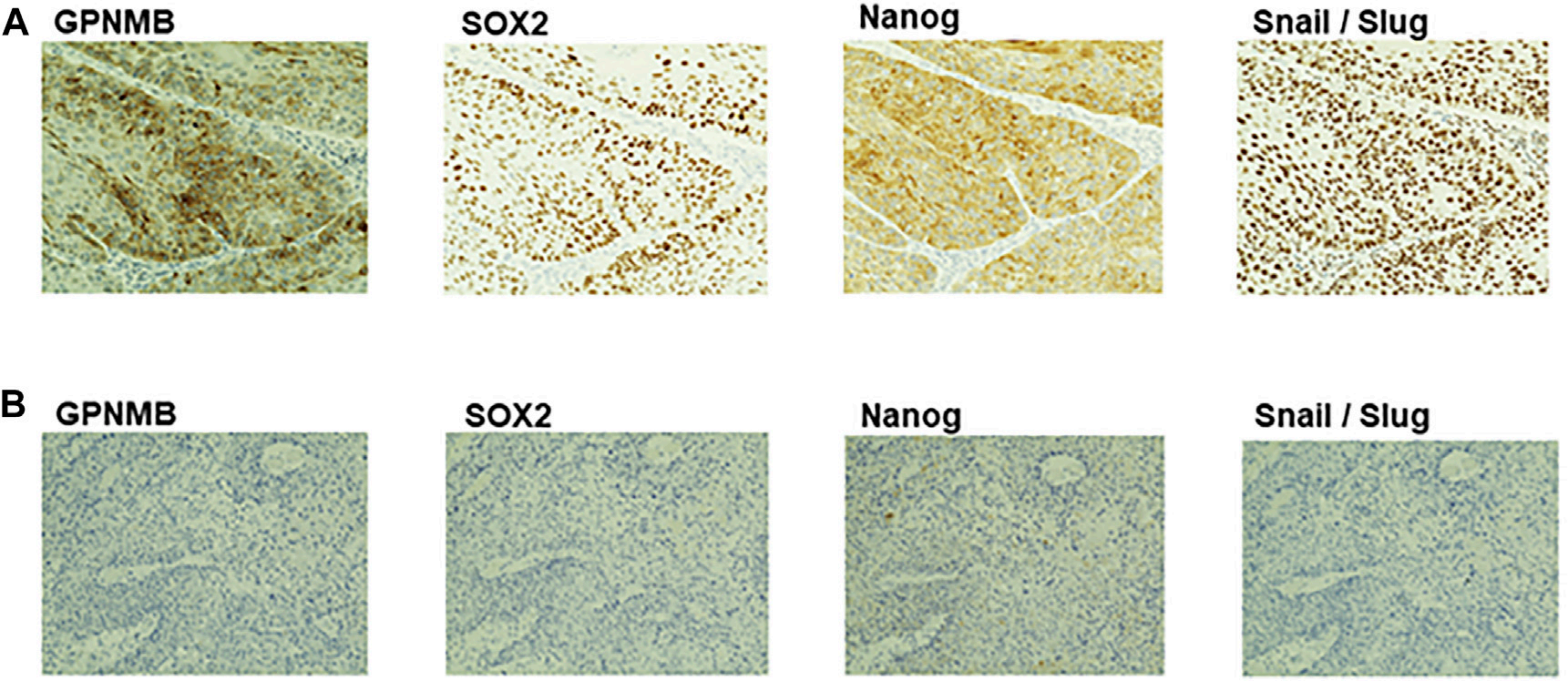
Immunohistochemical analysis of GPNMB, CSC markers, and EMT marker in serial sections of biopsy specimens. Serial sections of biopsy specimens were immunostained with the anti-GPNMB, anti-SOX2, anti-Nanog, and anti-Snail/Slug antibodies. Here demonstrated are two representative cases, one is an HNSCC expressing a high level of GPNMB **(A)**, the other is an HNSCC expressing a low level of GPNMB **(B)**. Note that the expression level of GPNMB is well correlated with those of CSC and EMT markers in serial sections of the same specimen. Magnification, ×200.

To compare the expression levels of GPNMB with those of CSC makers and EMT markers on the same specimens, we immunostained serial sections of biopsy specimens with the anti-GPNMB antibody along with the antibodies against CSC markers including SOX2 and Nanog and the antibody against EMT markers including Snail/Slug. As shown in Figure 8, while the HNSCC expressing a high level of GPNMB gave intensive signals of both CSC markers and EMT markers, that expressing no or a low level of GPNMB did not. This result thus indicates that GPNMB behaves as a marker of both CSC and EMT.

### Sphere Formation, Invasion, and Migration Manifested by GPNMB-Positive Cells Are Cancelled by the Inhibitor of ERK, an Effector of GPNMB

**FIGURE 9:**
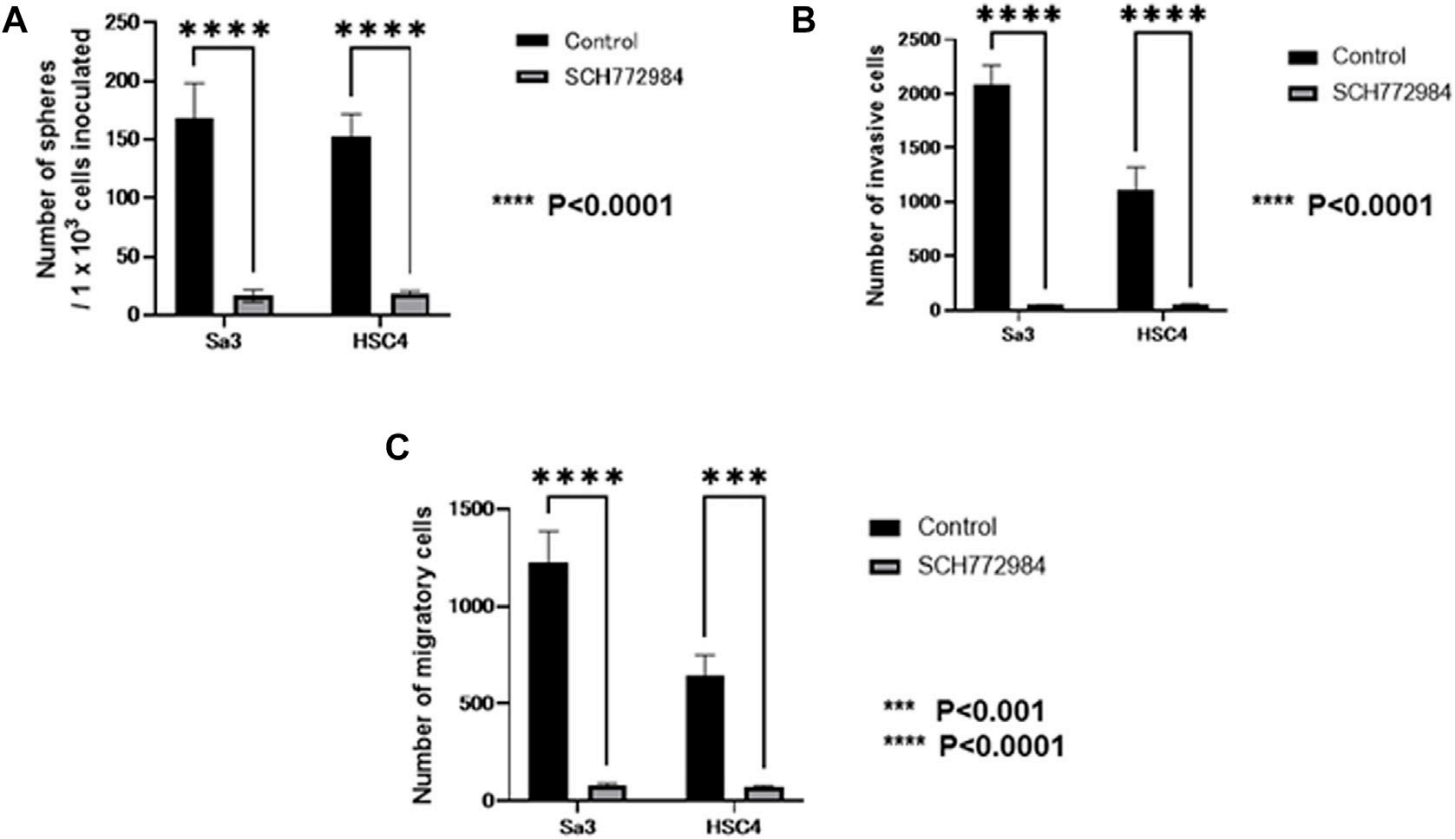
Effects of the ERK inhibitor on CSC and EMT characteristics manifested by GPNMB-positive cells. After FACS isolation of GPNMB-positive cells, CSC sphere formation, invasion, and migration assays were performed in the presence or absence of the ERK inhibitor. For control, DMSO only was added. **(A)** The number of CSC spheres formed by GPNMB-positive cells was greatly reduced by the ERK inhibitor. **(B)** Invasion assay of GPNMB-positive cells. **(C)** Migration assay of GPNMB-positive cells.

It is known that function of GPNMB is mainly mediated by ERK signaling. Then, we examined whether the ability of sphere formation, invasion, and migration is affected by an ERK inhibitor in GPNMB-positive cells. After FACS-isolation of GPNMB-positive fraction from each of Sa3 and HSC4 cell lines, GPNMB-positive cells were subjected to sphere formation, invasion, and migration assays in the presence or absence of the ERK inhibitor SCH772984. All of Sphere-forming ability (Figure 9A), invasive potential (Figure 9B), and migratory potential (Figure 9C) were strongly suppressed (*p* < 0.0001), suggesting that sphere formation, invasion, and migration enhanced in GPNMB-positive cells (Figures 2–4) should be mediated by GPNMB.

## Discussion

In this study, we found that GPNMB is expressed in HNSCC and that GPNMB-positive cells have enhanced sphere formation, invasion, and migration ability. The clinical specimens with high GPNMB expression had a poor prognosis and were resistant to chemoradiotherapy and bioradiotherapy. In the cases with lymph node metastasis, the GPNMB-positive cells were more concentrated in the lymph nodes than in the primary tumor.

When GPNMB-positive cells form spheres in breast cancer, the expression levels of CSC markers such as SOX2, NANOG, OCT4, CD44, CD133, and FOXO3 are elevated. It has been reported that these spheres have the characteristics of CSCs (21). GPNMB-positive cells in breast cancer also show an increased expression of EMT-related genes such as SNAIL, SLUG, and ZEB1, consistent with the fact that GPNMB-positive cells in HNSCC have shown increased invasiveness and migration (21). However, there are numerous theories about the mechanism of metastasis. It has been reported that matrix metalloproteinase-2 and matrix metalloproteinase-9 are involved in tumor invasiveness (22). With respect to metastasis, melanoma uses the GPNMB pathway to increase metastatic success by secreting soluble GPNMB, which binds to the vascular niche of DHL + endothelial cells. This binding has been reported to awaken angiogenic potential and promote tumor cell migration by excluding T cells from the niche (23). Thus, there are various theories that GPNMB activates the extracellular signal-regulated kinase signaling pathway (24), expresses matrix metalloproteinases (22, 25), and is associated with angiogenesis (23), which lead to metastasis; however, no consensus has been reached. In our present study on HNSCC, while GPNMB-positive cells formed CSC spheres in serum-free semisolid medium, GPNMB-negative cell formed nearly no sphere (Figure 2). It is thus likely that GPNMB-positive cells have CSC characteristics in HNSCC. Furthermore, immunostaining showed that the expression level of GPNMB in HNSCC is correlated with that of the CSC markers and the EMT markers (Figure 8). Elucidating the mechanism of metastasis in HNSCC is an important step in the search for preventing this cancer.

We immunostained GPNMB biopsy specimens to investigate the association between OS and PFS, and cases with high GPNMB expression had a very poor prognosis. The same result has been reported for various carcinomas, such as hepatocellular carcinoma (26), epithelial ovarian carcinoma (27), glioma (28), and breast cancer (29); HNSCC was no exception. In renal cell carcinoma, GPNMB expression is closely related to bone metastasis, which is a poor prognostic factor (30); in terms of poor prognostic factors, they are the same as ours. GPNMB expression for distant metastasis should also be investigated in HNSCC. Interestingly, the high GPNMB expression group was resistant to chemoradiotherapy and bioradiotherapy and had a very poor prognosis. If GPNMB-positive cells have CSC characteristics and can induce EMT, this could explain their resistance to chemoradiotherapy and bioradiotherapy (31). We also compared the intensity of primary and metastatic lymph nodes by immunostaining them with GPNMB in a case of actual lymph node metastasis and found that GPNMB-positive cells were more concentrated in the metastatic lymph nodes than in the primary tumor. There have been no reports of comparative GPNMB expression between primary and metastatic lymph nodes in other carcinomas, and the cause of this is unknown; however, we believe this is important evidence that GPNMB is related to EMT.

Glembatumumab vedotin is currently being tested in clinical trials as a GPNMB-targeted therapy and is being tested for clinical use in breast cancer, malignant melanoma, and recurrent osteosarcoma (32–34).

In HNSCC, we will continue to analyze the function of GPNMB to establish a new therapeutic strategy targeting GPNMB and to improve prognosis. Results from clinical specimens suggest that GPNMB can be used as a marker of radiosensitivity and contribute significantly to individual treatment decisions.

## Conclusion

GPNMB-positive cells had enhanced sphere formation, invasion, and migration ability compared with GPNMB-negative cells. As reported in breast cancer, GPNMB-positive cells might have CSC characteristics and the ability to induce EMT. When clinical specimens were immunostained with GPNMB and examined for their association with OS and PFS, the high GPNMB expression group had a poor prognosis. The high GPNMB expression group was also resistant to chemoradiation therapy. GPNMB is more highly expressed in metastases than in primary tumors, suggesting that GPNMB plays an important role in HNSCC; further functional analyses will help improve its prognosis. In the future, we would like to increase the sample size, unify the chemotherapy regimen, validate GPNMB *in vivo*, and evaluate EMT and CSC markers to establish a new treatment targeting GPNMB.

## Data Availability

The raw data supporting the conclusion of this article will be made available by the authors, without undue reservation.

## Abbreviations

CI, Confidence interval; HNSCC, Head and neck squamous cell carcinoma; HR, Hazard ratio; GPNMB, glycoprotein (transmembrane) nmb/glycoprotein nonmetastatic melanoma protein B; OS, Overall survival; PFS, Progression-free survival.
